# Diabetes, Hypertension, Atrial Fibrillation and Subsequent Stroke-Shift towards Young Ages in Brunei Darussalam

**DOI:** 10.3390/ijerph19148455

**Published:** 2022-07-11

**Authors:** Burc Bassa, Fatma Güntürkün, Eva Maria Craemer, Uta Meyding-Lamadé, Christian Jacobi, Alp Bassa, Heiko Becher

**Affiliations:** 1 Department of Neurology, Krankenhaus Nordwest, 60488 Frankfurt am Main, Germany; bassa.burc@khnw.de (B.B.); craemer.eva.maria@khnw.de (E.M.C.); meyding-lamade.uta@khnw.de (U.M.-L.); jacobi.christian@khnw.de (C.J.); 2 Center for Biomedical Informatics, University of Tennessee Health Science Center, Memphis, TN 38103, USA; fgunturk@uthsc.edu; 3 Department of Mathematics, Bogazici University, 34342 Istanbul, Turkey; alp.bassa@gmail.com; 4 Institute of Medical Biometry and Epidemiology, Medical Center Hamburg-Eppendorf, Martinistr. 52, 20246 Hamburg, Germany

**Keywords:** stroke, Southeast Asia, Brunei Darussalam, stroke-epidemiology, stroke risk factors

## Abstract

Southeast Asia harbors a young population of more than 600 million people. Socioeconomic transition within the last decades, driven by globalization and rapid economic growth, has led to significant changes in lifestyle and nutrition in many countries of this region. Hence, an increase in the number of non-communicable diseases is seen in most populations of Southeast Asia. Brunei Darussalam is the smallest country in this region, with a population of around 400,000 inhabitants. Vast hydrocarbon resources have transformed Brunei into a wealthy industrialized country within the last few decades. We compared the age distribution and prevalence of cardiovascular risk factors in ischemic stroke patients between the only stroke unit in Brunei Darussalam and a tertiary stroke center from Frankfurt/Germany. Between 2011 and 2016, a total number of 3877 ischemic stroke patients were treated in both institutions. Even after adjusting for age due to different population demographics, stroke patients in Brunei were younger compared to their German counterparts. The prevalence of hypertension and diabetes mellitus was significantly higher in young age groups in Brunei, whereas no difference was observed for older patients. The rapid socioeconomic transition might be a significant risk factor for the development of non-communicable diseases, including stroke.

## 1. Introduction

Despite the merited public attention to infectious diseases in view of the recent SARS-CoV-2 pandemic, non-communicable diseases (NCD) remain the most important cause of death and disability worldwide. In 2016, an estimated 40.5 million people died from NCD or their complications, accounting for more than 70% of overall mortality. The World Health Organization (WHO) projects a dramatic increase in the rate, mortality, and morbidity of NCD in many regions, including Southeast Asia over the next few decades, so that half of all deaths in this region will be due to non-communicable diseases [1,2,3].

Almost 80% of the mortality and morbidity of non-communicable diseases are caused by cardiovascular diseases, including myocardial infarction and stroke. These diseases share common risk factors, most notably raised blood pressure, high body-mass index (BMI), elevated blood sugar levels, and an abnormal serum lipid concentration. All four conditions are on the rise in Southeast Asia [4].

Although, historically, the prevalence of cardiovascular risk factors such as hypertension, diabetes, and hyperlipidemia has been low in Southeast Asia, recent studies show an opposite trend, especially for younger age groups. Despite a global decrease in average blood pressure, an increasing trend is observed in many Southeast Asian countries [5,6].

The underlying reasons for this development need further investigation, but the significant socio-economic development in many Southeast Asian countries over the recent decades seems to play a pivotal role. Many countries in this region are currently in a state of nutritional transition, driven by rapid economic growth and globalization. Progressively, the traditional diet in this region, rich in fibers and fruits, is being displaced by high-energy, processed foods. With increasing wealth and purchasing power in larger parts of the population, there is an increasing transition towards a “Western diet”, which is characterized by energy-dense foods containing high amounts of protein and fat [7,8].

Increasing physical inactivity, a well-known consequence of progressive urbanization, in conjunction with high energy food consumption, have led to significant lifestyle changes, ultimately resulting in an increased BMI. The average BMI, blood lipids, and blood pressure also increase, eventually leading to the increased NCD incidence [9,10,11,12].

Brunei Darussalam is a country on the northern shores of Borneo Island, in Southeast Asia. Due to its natural resources, it underwent a rapid economic transition over the recent decades. A population-based epidemiological study conducted in 2014, in which blood pressure measurements were conducted as part of the study protocol, revealed a high prevalence of arterial hypertension, especially in the younger age groups of the Bruneian population [5]. The healthcare system in Brunei is completely financed by the government. Thus, every Bruneian citizen has free access to health care. A small number of private outpatient clinics are present, but these clinics only offer specialized care to a very minor part of the population.

An association between a higher prevalence of cardiovascular risk factors in younger individuals and the development of cardiovascular disease at an earlier age was shown for similar countries [13].

Germany, on the other hand, is an industrialized country which started strong economic growth after the second World War. Over the last decades, its cardiovascular and cerebrovascular disease incidence and mortality rates have been stable or have decreased, similarly to its neighboring countries in Europe [6,7,8,9].

Between the years 2010 to 2019, a cooperation between Krankenhaus Nordwest (KHNW) in Frankfurt Germany and the Bruneian government existed with the scope to set up a stroke unit in Brunei Darussalam [14].

The Brunei Neuroscience Stroke and Rehabilitation Center (BNSRC) was established in 2010, being the first stroke unit of Brunei Darussalam. Identical stroke treatment protocols were implemented in both institutions. A team consisting of a consultant neurologist, two neurological registrars and a nurse specialized in the care of stroke patients from Krankenhaus Nordwest was continuously onsite in Brunei Darussalam, assisting the local team consisting of doctors and nurses. Furthermore, Bruneian doctors and nurses rotated to the stroke unit in Krankenhaus Nordwest.

Neuroradiological imaging studies conducted in Brunei Darussalam were transferred to Frankfurt Germany through a dedicated line. Neuroradiologists from Frankfurt Germany reported these studies and gave feedback to the local doctors.

There is no systematic analysis of cerebrovascular risk factors in Bruneian stroke patients at present. We hypothesized that the stroke incidence of the younger population in Brunei Darussalam is higher than in a typical western industrialized country, while the incidence in the older population may be similar or lower. We analyzed the age distribution of ischemic stroke patients, as well as the distribution of selected cardiovascular risk factors in Brunei Darussalam.

## 2. Materials and Methods

All patients with a CT- or MRI-proven ischemic first ever or recurrent stroke treated between 2011 and 2016 at the Brunei Neuroscience Stroke and Rehabilitation Centre in Brunei Darussalam were included. Data on sex, age of stroke onset, and the prevalence of the stroke risk factors arterial hypertension, diabetes mellitus, and atrial fibrillation in acute ischemic stroke (AIS) were retrieved from medical records. The same data were retrieved from patients treated at Krankenhaus Nordwest in Frankfurt/Germany from the local stroke registries.

The Brunei Neuroscience Stroke and Rehabilitation Centre was established in 2010 as a cooperation between the Government of Brunei Darussalam and the Neurological Department of Krankenhaus Nordwest. It is the only stroke unit in the country, so virtually all patients with ischemic stroke are admitted or transferred to this unit for further management [14].

Krankenhaus Nordwest is a high-volume stroke center in the state of Hesse, Germany. The age distribution of stroke patients, as well as the prevalence of cardiovascular risk factors, are representative of the state of Hesse as well as other stroke units in Germany [15].

Patients with a transient ischemic attack or hemorrhagic stroke were excluded from the analysis. 

All data were collected in an anonymized fashion from a database created for quality control purposes. In Germany, the stroke registry is based on state law for quality assurance measurement, and the registration is mandatory for all patients with stroke in the state of Hesse, Germany. All patients were registered anonymously, as published previously. Neither institutional board approval nor informed consent was required [16,17].

Similarly, in Brunei Darussalam, data was collected in an anonymized fashion to ensure the fulfillment of quality-control requirements during the setup of the unit [14].

Frequencies, percentages, mean, and standard deviation of risk factors were calculated for both patient groups by sex and 5-year age groups. Prevalence rates for each risk factor by age and sex were calculated and compared for both countries. Confidence intervals for PRRs (prevalence rate ratio) were calculated using the normal approximation. Since the population age distribution significantly differs between Brunei Darussalam and Germany, the population percentages by age group and sex were obtained from the population registers and used for standardization. We calculated the standardized mean age of cases in Brunei Darussalam, standardized to the population distribution in Germany, and compared it to the mean age of cases in the German case group.

For the analyses, the statistical software program R 3.5.2 (R foundation, Vienna, Austria) was used.

## 3. Results

Between the years 2011 to 2016, 845 patients with AIS were treated at the Brunei Neuroscience Stroke and Rehabilitation Centre compared to 3032 stroke patients treated in Krankenhaus Nordwest/Germany. The mean age of AIS patients in Brunei was 61.2 years (62.8 and 60.3 years in female and male patients, respectively), which increased to 71.1 years (73.9 years for female patients and 68.6 years for male patients) after age standardization to a German reference population. Stroke patients from Germany were older with a mean age of 73.8 years (76.8 and 70.9 years for female and male patients, respectively). 

**Figure 1:**
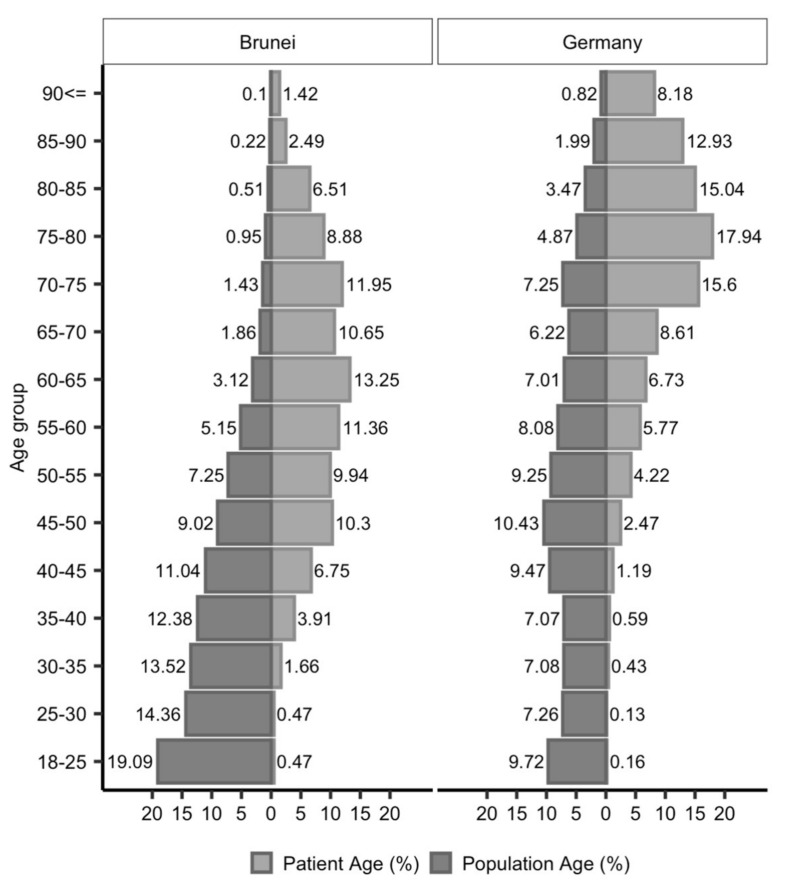
The Age Distribution of Stroke Patients in Conjunction with Demographic Population Characteristics.

Thus, even after adjusting for differences caused by the much younger population in Brunei Darussalam, Bruneian stroke patients remained significantly younger compared to their German counterparts (71.1 vs. 73.8, difference 2.7 years, 95% confidence interval [1.6, 3.8]). The age distribution of stroke patients in conjunction with demographic population characteristics is summarized in Figure 1.

Younger individuals in Brunei Darussalam show a higher stroke incidence compared to patients in Frankfurt/Germany, whereas the stroke rate seems to be higher in older patients in Frankfurt/Germany.

### 3.1. Prevalence of Stroke Risk Factors

The distribution of demographics and risk factors of stroke patients were summarized in Table 1 for both countries. PRR (risk in Brunei/risk in Germany) for each risk factor by age and sex were generated to determine whether the distribution of these risk factors differs between the two patient populations.

#### 3.1.1. Arterial Hypertension

**Figure 2:**
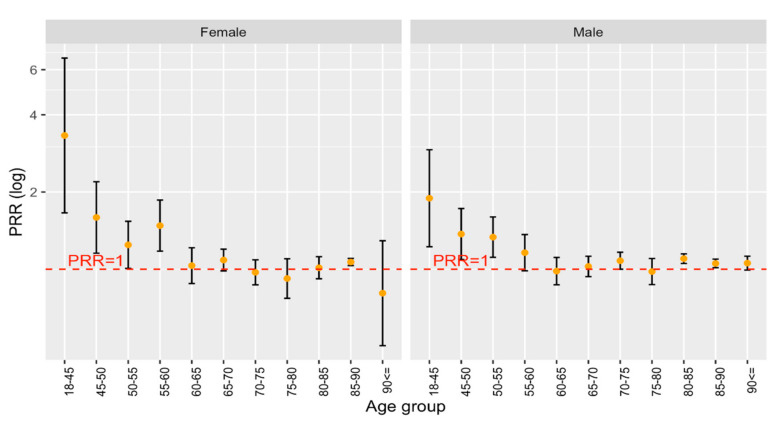
PRR for Arterial Hypertension by Age group and Sex in Stroke Patients.

Arterial hypertension was the most prevalent risk factor among all stroke patients. Hypertension is generally considered the most important modifiable risk factor for cerebrovascular diseases [18]. Overall, 85.72% of all German stroke patients were classified as hypertensive (87% of all female and 84.5% of all male patients). In comparison, 86.75% of all Bruneian patients were classified as hypertensive (87.9% of all female and 86% of all male stroke patients). After age and sex standardization to a German reference population, this number increased to 91.08% (90.7% of all female and 91.7% of all male patients). As shown in Figure 2, the PRR of arterial hypertension is higher than the one among younger patients and close to the one among older age groups. This result is compatible with previous studies, which already showed that the prevalence of arterial hypertension in the general Bruneian population is higher compared to most European countries and Malaysia [5].

#### 3.1.2. Diabetes Mellitus

**Figure 3:**
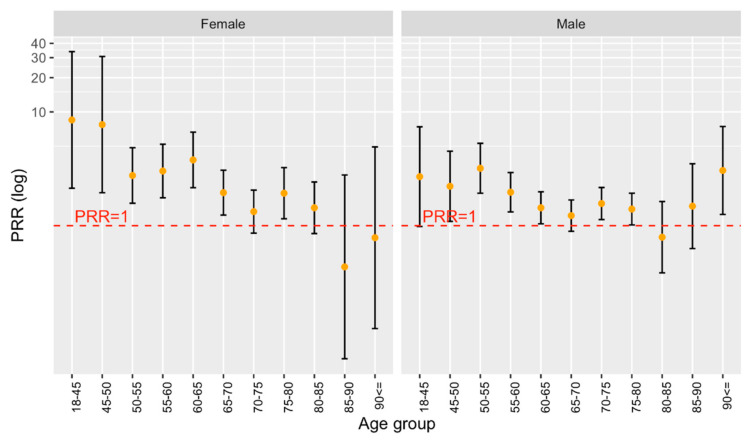
PRR for Diabetes Mellitus by Age group and Sex in Stroke Patients.

Overall, 26.45% of all stroke patients treated in Germany were diagnosed as being diabetic (22.84% of female and 29.9% of male patients). In comparison, 46.98% of all Bruneian stroke patients were diabetic (51.39% of females and 44.25% of males). In younger Bruneian stroke patients, the prevalence of diabetes was significantly higher compared to their German counterparts, although for females the pattern is not as clear (Figure 3). The data available did not allow a distinction between type 1 and type 2 diabetes.

#### 3.1.3. Atrial Fibrillation

**Figure 4:**
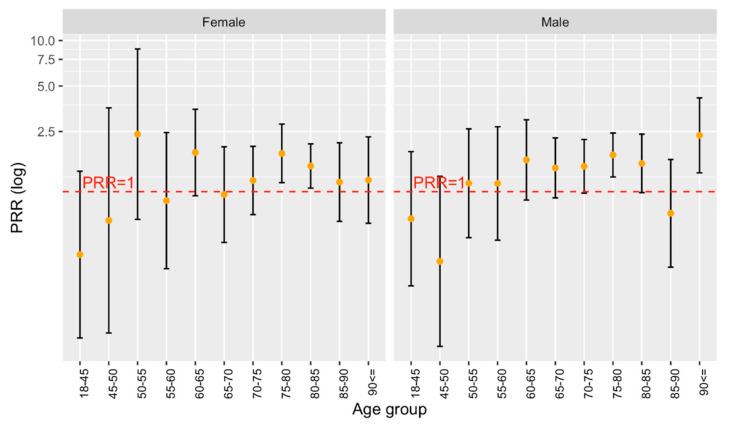
PRR for Atrial Fibrillation by Age group and Sex in Stroke Patients.

Overall, 29% of all German stroke patients had an existing or newly diagnosed atrial fibrillation (32.97% of females and 25.19% of males) and 25.3% of all Bruneian patients had an atrial fibrillation (27.55% of all female and 23.95% of all male patients). The prevalence of atrial fibrillation is, on average, slightly higher in Bruneian stroke patients, compared to stroke patients from Germany, for both sexes, with no clear age trend (Figure 4).

**Table 1:** The distribution of demographics and risk factors in German and Bruneian stroke patients.

	FEMALE					MALE				
	German		Bruneian			German		Bruneian		
	N	%	N	%	% *	N	%	N	%	% *
Sex	1480	48.81	323	38.22	43.94	1552	51.19	522	61.78	56.06
Sex 18+ **		51.7		48.3			48.3		51.7	
Age Groups										
18–40	21	1.42	19	5.88	0.9	19	1.22	36	6.90	1.4
40–45	13	0.88	19	5.88	1.5	23	1.48	38	7.28	2.4
45–50	29	1.96	30	9.29	2.9	46	2.96	57	10.92	4.7
50–55	36	2.43	30	9.29	3.3	92	5.93	54	10.34	4.9
55–60	53	3.58	38	11.76	5.0	122	7.86	58	11.11	6.6
60–65	69	4.66	38	11.76	6.9	135	8.70	74	14.18	12.2
65–70	99	6.69	29	8.98	8.3	162	10.44	61	11.69	13.8
70–75	197	13.31	45	13.93	19.4	276	17.78	56	10.73	21.4
75–80	270	18.24	28	8.67	12.2	274	17.65	47	9.00	16.7
80–85	247	16.69	31	9.60	23.7	209	13.47	24	4.60	9.3
85–90	254	17.16	9	2.79	9.8	138	8.89	12	2.30	4.8
90<=	192	12.97	7	2.17	6.1	56	3.61	5	0.96	1.8
Risk Factors										
HT	1287	86.96	284	87.93	90.67	1312	84.54	449	86.02	91.67
Diabetes	338	22.84	166	51.39	34.05	464	29.90	231	44.25	42.28
AF	488	32.97	89	27.55	45.14	391	25.19	125	23.95	40.91
	$\bar{x}$	sd	$\bar{x}$	sd	$\bar{x}$ *	$\bar{x}$	sd	$\bar{x}$	sd	$\bar{x}$ *
Age	76.8	12.8	62.8	15	73.9	70.9	12.5	60.3	13.8	68.6
Age 18+ **	51.2	19.1	38.2	14.4		48.7	17.8	37.7	14	

* Age and sex standardized to German population ** Population older than 18 years of age.

## 4. Discussion

The healthcare systems of most countries in Southeast Asia are structured to deal with the acute nature of infectious diseases, which had the highest impact on population health until a few decades ago. In many regions, the infrastructure is insufficient to cope with the chronic nature of non-communicable diseases, which require long-term patient monitoring and hence are expensive and workforce-intensive [19].

In our study, the incidence of AIS was higher in younger age groups in Brunei Darussalam compared to patients from Frankfurt/Germany.

Furthermore, in younger age groups, Bruneian stroke patients had a significantly higher prevalence of hypertension and diabetes mellitus. Interestingly, this difference was not observed in older age groups, in which the rates were comparable. The prevalence of atrial fibrillation was similar throughout all age groups in both populations.

Our study lines up with multiple previous studies from neighboring countries, showing a high prevalence of cardiovascular risk factors, especially in younger individuals in East and Southeast Asia. A study from Malaysia including only young stroke patients showed that the prevalence of hypertension and diabetes mellitus was significantly higher compared to Australian stroke patients with a similar age distribution [20].

A large-scale study from India showed that South Asians have high rates of acute myocardial infarction (AMI) at younger ages compared with individuals from other countries. This was mainly attributed to the high prevalence of cardiovascular risk factors in younger age groups [21].

Hypertension is the most important and prevalent stroke risk factor. There is an alarming increase in the prevalence of hypertension in most Asian countries. During the past four decades, the highest worldwide blood pressure levels have shifted from high-income countries in Europe and the US to South Asia and sub-Saharan Africa [22].

Stroke incidence correlates with systolic and diastolic blood pressure levels in the population. In Brunei Darussalam, the blood pressure levels among the younger individuals were higher compared to same ages individuals from neighboring countries and Europe [5,23].

Other risk factors for NCD are on the rise in most Asian countries. In recent years, a pronounced increase in the prevalence of diabetes mellitus has been observed in most of the Asian countries, corresponding to an increase in the levels of BMI [24].

While Southeast Asia has some of the lowest prevalence of overweight and obesity globally, there has been an alarming increase in the recent decade, with an estimate of 6.6 million young children under 5 years of age and about 20% of the adult population being currently overweight [25].

The prevalence of diabetes has doubled in the US population in the past 40 years. Although this development is grave, the prevalence has tripled in most Asian countries within the same time period. Despite a significant heterogeneity, in most Asian populations diabetes develops in a much shorter time, in a younger age group, and in people with much lower BMI [26,27].

There are abundant theories to explain the disparity in the susceptibility to cardiovascular disease, including a higher tendency of abdominal obesity and the earlier development of impaired glucose metabolism in populations with a certain genetic background, but the exact underlying mechanisms still remain unknown.

One of the most interesting theories from the evolutionary standpoint is the thrifty gene hypothesis, stating that genetic variations favoring energy storage during times of famine were positively selected. Now, with easier access to high-energy food and an increasingly sedentary lifestyle in most of these populations, these genetic traits might have become nonbeneficial [28,29].

As a limitation of our study, we would like to mention that a direct comparison of incidence rates in Brunei and Germany was not possible since the German case series is hospital-based and does not allow us to define the underlying population. Therefore, the denominator to calculate incidence rates is not available. There are several hospitals in the Frankfurt area with stroke units, and the coverage regions overlap. We are sure, though, that the stroke cases are not selected with respect to the factors considered here, but rather a representative group. Another limitation is that in both case series we do not have information on whether the disease was a first ever or a recurrent stroke.

No significant difference in the prevalence of atrial fibrillation was observed between both patient groups in any age group. Although further studies are needed for the proper interpretation of this result, multiple previous studies indicate that South Asians may have a reduced risk of developing atrial fibrillation, despite having a higher prevalence of traditional cardiovascular risk factors [30,31].

This study has several limitations. Although the official strategy of the Bruneian Ministry of Health during the analyzed period was to transfer all stroke patients to BNSRC for further treatment and management, we cannot rule out that some patients, especially with very severe or light symptoms, were treated locally and hence not included in this analysis. Furthermore, the demographics and risk factors of patients from Frankfurt Germany are representative for the State of Hesse but might not be representative for all stroke units in Germany.

## 5. Conclusions

Southeast Asia inhabits a young population of more than 600 million people. It is a heterogeneous region in means of demographic characteristics, economic development, and lifestyle. Brunei is economically stronger than its neighboring countries, so these results might not be representative for all countries. However, the effect of stroke at young ages in Brunei Darussalam, and possibly other affected countries in this region, is significant. In addition to the severe and sometimes catastrophic burden of stroke in young ages on an individual level, the social impact, as well as the burden on healthcare systems, is immense. Long-term treatment costs and loss of workforce are just some of the consequences. Increasing the awareness on non-communicable diseases in conjunction with the implementation of preventive public health policies are essential steps to mitigate future effects of non-communicable diseases in this region.

## Data Availability

All data analyzed are available from the corresponding author on reasonable request.
